# Supplementary material for: Chromothripsis during telomere crisis is independent of NHEJ, and consistent with a replicative origin
Source: Genome Res. 2019 May;29(5):737–49. doi: 10.1101/gr.240705.118 (PMC6499312; doi:10.1101/gr.240705.118)
Supplement: Supplemental Material [file supp_gr.240705.118_Supplemental_file_1.zip › contigs/annotated_contigs/DB92/contig.2.DB92_length_382_mean_cov_7.06806282723.docx]

**DB92_length_382_mean_cov_7.06806282723**

```
CAGAGGCATCTGTGGCTGGTATCTGGAACTTTGTGACTCTAAACCAAACTCTCCCCCATCTTTTTATCTCCAACTTTTCCGAAAGGGTT
 >chrX:11351885-11352165 - E=3e-157
TACATGTGTGAGAAACTCATAGCACCAAAAAACTTGTCAATTATGCCACTGCAATATTTCAGAAGCTTTAACTGAAGCATCTTACGGTG

TCAGGGGTTCTTCATGAGAAATAGCACACATTAGAGGCTTTGAGAGAAGACCTAGTTCTGTCATTAACAGTCAGCCTCAAGTTTGTCCT

CCCATGATGG|AGC|TTTTAATCAATTGGCATGGACCAATTCTAATCAATTGAGTCAATATTTATTAAGCACACTCTAGGGCAAGGCAC
           >chrX:11319774-11319879 + E=1e-51
TATTCCATGTGCTGGTGAAAAGAGCAAG
```
